# Genome Analysis of Rift Valley Fever Virus, Mayotte

**DOI:** 10.3201/eid1806.110994

**Published:** 2012-06

**Authors:** Catherine Cêtre-Sossah, Hervé Zeller, Marc Grandadam, Valérie Caro, François Pettinelli, Michèle Bouloy, Eric Cardinale, Emmanuel Albina

**Affiliations:** Centre de Coopération Internationale en Recherche Agronomique pour le Développement, Montpellier, France (C. Cêtre-Sossah, E. Albina);; Institut Pasteur, Paris, France (H. Zeller, M. Grandadam, V. Caro, M. Bouloy);; Centre Hospitalier de Mayotte, Mayotte (F. Pettinelli);; Centre de Coopération Internationale en Recherche Agronomique pour le Développement, Sainte-Clotilde, France (E. Cardinale)

**Keywords:** Rift Valley fever, Rift Valley fever virus, arbovirus, Mayotte, viruses, zoonoses, genome analysis

## Abstract

As further confirmation of a first human case of Rift Valley fever in 2007 in Comoros, we isolated Rift Valley fever virus in suspected human cases. These viruses are genetically closely linked to the 2006–2007 isolates from Kenya.

Identified during the 1930s in Kenya, Rift Valley fever (RVF) is a zoonotic disease that circulates in many African countries and in the Arabian Peninsula (*1**,**2*). RVF virus (RVFV) epizootics are characterized by large sweeping abortion storms and substantial death rates in adult livestock (primarily sheep, goats, and cattle), with the death rate for newborn animals approaching 100% (*3*). Humans typically are infected by bites of infected mosquitoes or by percutaneous or aerosol exposure to contaminated fluids from infected animals. In most human cases, RVF is characterized by a self-limiting febrile illness (2–5 days), which progresses to more serious complications (hepatitis, encephalitis, blindness, or hemorrhagic syndrome) in only 1%–2% of infected persons (*4**,**5*). A large epizootic–epidemic occurred during 2006–2007 on the eastern African mainland, predominantly in Kenya (*6*) and Madagascar, during 2 successive rainy seasons (*7*).

In July 2007, a 12-year-old boy with a 2-month history of severe encephalitis was transferred from the Grande Comore, Union of the Comoros, to Mayotte (*8**,**9*). RVF infection was confirmed by IgM serologic analysis. Because of the proximity of Comoros and Mayotte, the RVF situation among humans in Mayotte was assessed. In serum samples from 7 humans with dengue-like syndromes, RVFV IgM or RVFV RNA was detected. We report the isolations and full sequence analysis of 2 RVF viral isolates from these serum specimens.

## The Study

During January–April 2007, seven patients native to Mayotte were admitted to the hospital for severe dengue-like syndromes. Two patients were RVF seropositive by IgM and IgG, and the other 5 were positive by RVFV-specific reverse transcription PCR (RT-PCR) as detailed in Sissoko et al. (*9*). As described for other viruses, we used in-house IgM-capture enzyme immunoassays and in-house direct detection for IgG by using microplates coated with RVFV antigen and specific binding by using a peroxidase-labeled goat anti-human IgG conjugate (*10*).

RVFV isolates were obtained on Vero E6 cells from the serum of 2 hospitalized patients (serum collected on February 21 and March 20, 2008). RNA extracted by using the RNaid Kit (Qbiogene, Carlsbad, CA, USA) was reverse transcribed by PCR and amplified by using SuperScript One-Step RT-PCR with platinum Taq kit (Invitrogen, San Diego, CA, USA) with primers targeting the small, medium, and large segments (adapted from [*11*]). Overlapping RT-PCR fragments were purified by ultrafiltration. Sequencing reactions were performed by using the Big Dye Terminator v1.1 cycle sequencing kit (Applied Biosystems, Foster City, CA, USA). Sequence chromatograms from both strands were obtained on automated sequence analyzer ABI3730XL (Applied Biosystems). For sequence analysis, contig assemblies and sequence alignments were performed by using BioNumerics version 6.6 (Applied-Maths, Sint-Martens-Latem, Belgium).

We used 2 methods for phylogenetic reconstruction: maximum likelihood and the Bayesian inference. The best models of nucleotide substitution for each dataset were selected from the uncorrected and corrected Akaike Information Criterion, the Hannan and Quinn performance-based decision theory and Bayesian Information Criterion of Jmodeltest version 0.1 and TREEFINDER version October 2008 (Munich, Germany, distributed by its author at www.treefinder.de). The consensus substitution models proposed by the different software packages were selected for further analyses. Comparison of the maximum-likelihood method implemented by the TREEFINDER program with others was performed on the small, medium, and large segments by using the neighbor-joining and maximum parsimony methods from Mega5 software and the Bayesian approach by using MrBayes version 3.0B4 for phylogenetic reconstruction with random starting trees and run for 2,000,000 generations, sampling the Markov chains at intervals of 100 generations (*12**,**13*). Branch support values were obtained by using nonparametric bootstrapping with 1,000 resampling for PhyML and TREEFINDER and the posterior probabilities for the Bayesian approach estimated on 10,000 samples (sample frequency set to every 100th generation by using the Markov Chain Monte Carlo sampling). We compared topologies of the maximum-likelihood and Bayesian trees obtained for the different segments.

The complete genome sequences performed on 2 human RVFV isolates from Mayotte referenced as 2008/00099 and 2008/00101 (deposited in GenBank/EMBL under accession nos. HE687302–HE687307) are embedded within the larger 2006–2007 East African clade, specifically within the lineage previously termed Kenya-1 (Table A1). The Kenya-1 virus lineage includes 18 isolates, 8 human isolates (035/07Baringo Kenya 2007, SPU10315 Kenya 2007, Garissa 004/006 Kenya 2006, Dod002/007Tanzania 2007, 3162 Madagascar 2008, 3163 Madagascar 2008, 3164 Madagascar 2008, 3165 Madagascar 2008), 2 mosquito isolates (KLFMsq091/07 Kenya 2007, 131B04/06Garissa Kenya 2006), and 8 livestock isolates (1602Mombassa Kenya 2007, 2820Garissa Kenya 2007, 3644Baringo Kenya 2007, 473Kajaido Kenya 2007, 0611Kenya 2007, 3168 Madagascar 2008, 3169 Madagascar 2008, 3170 Madagascar 2008). The Kenya 2 virus lineage comprises 3 human isolates (1811Garissa Kenya 2006, 0094 Kenya 2007, Tan001/007 Tanzania 2007).

Because maximum-likelihood and Bayesian tree topologies obtained for the 3 segments were similar, only the small segment is presented. The Figure shows the Bayesian tree topology based on all RVFV small segments, with the HB29 phlebovirus from the People’s Republic of China as an outgroup. Tree topologies are consistent with those generated in previous work (*7**,**11*). The reliability of the phylogenetic trees was confirmed by performing bootstrap analysis. The Kenya 1 and Kenya 2 lineages clustered together with an overall bootstrap value of 92% but with sublineage bootstrap values of 56%–100%.

**Figure:**
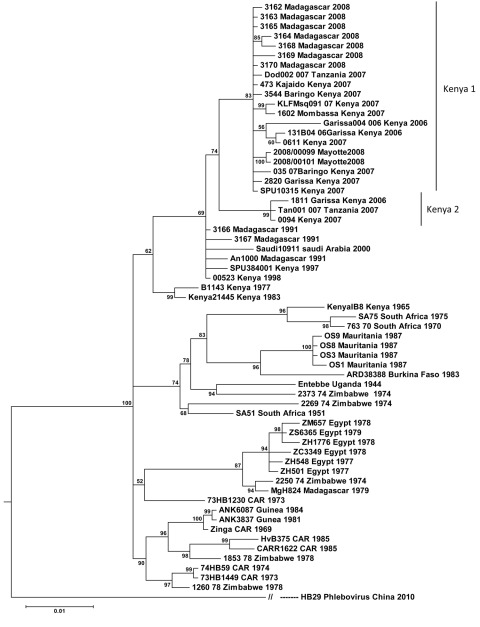
Fifty-two complete sequences of Rift Valley fever virus small genome segments aligned and analyzed by the Bayesian program (MrBayes). Scale bar indicates nucleotide substitutions per site.

## Conclusions

The work of Sissoko et al. (*9*) suggested the indigenous transmission of RVFV in humans in Mayotte. The geographic distribution of the 10 human serum samples found positive for RVFV in 2007 and 2008 was not spatially delimited. All case-patients were native to the island and resided in the following districts: Mamoudzou (3), Brandaboua (2), Dembeni (1), Sada (1), Chirongui (2), and Boueni (1). None reported travel into countries where RVF is endemic (*9*). The genomic analysis of the Mayotte isolates placed them within the 2006–2007 eastern African Kenya-1 lineage. RVF activity in Mayotte appears to be an expansion of the eastern African mainland 2007–2008 outbreak. It illustrates the risk for introduction in Mayotte or other Comorian islands of infectious agents involved in outbreaks in neighboring eastern African coastal countries, the major source being livestock importation from the African mainland or Madagascar.

The recent data published on RVFV Malagasy strains (*7**,**14*) support an epidemic cycle with introduction of the virus from outbreaks on mainland eastern Africa rather than an enzootic cycle in Madagascar. RVFV has been isolated from at least 40 species of mosquitoes in 8 genera. Recent experimental RVFV infections on African mosquito species revealed that 8 species—*Aedes palpalis* (Newstead), *Ae. mcintoshi* Huang, *Ae. circumluteolus* (Theobald), *Ae. calceatus* Edwards, *Ae. aegypti* (L), *Culex antennatus* (Becker), *Cx. pipiens* (L), and *Cx. quinquefasciatus* Say—are susceptible to infection, and that all except *Ae. calceatus, Ae. aegypti*, and *Cx. quinquefasciatus* transmitted RVFV by bite after oral exposure (*15*). In Mayotte, a preliminary study has shown that 4 species—*Ae. circumluteolus*, *Cx. antennatus*, *Cx. quinquefasciatus*, and *Ae. aegypti*—are present (T. Balenghien, V. Robert, pers. comm.).

Even if mosquito transmission might have occurred among some of the 7 reported RVF case-patients, contact with imported ruminants is the predominant means of exposure among these reported case-patients. However, further entomologic studies need to be conducted to identify all potential vector species in the island and animal surveys need to be conducted to help detect RVF at early stages to gain a better understanding of the ecologic and climatic factors that favor RVFV dissemination. These assessments will help in the development of appropriate control measures to better predict and respond to potential RVF outbreaks.

## Appendix

**Table A1:** Strains analyzed in study of the genome analysis of Rift Valley fever virus, Mayotte

Source of isolate	Virus strain	Country of origin	Year isolated	GenBank accession no. for small segment
Bovine	763/70	Zimbabwe	1970	DQ380174
Bovine	1260/78	Zimbabwe	1978	DQ380164
Bovine	1853/78	Zimbabwe	1978	DQ380168
Bovine	2250/74	Zimbabwe	1974	DQ380143
Bovine	2269/74	Zimbabwe	1974	DQ380173
Bovine	2373/74	Zimbabwe	1974	DQ380159
Human	73HB1230	Central African Republic	1973	DQ380172
Human	73HB1449	Central African Republic	1973	DQ380162
Human	74HB59	Central African Republic	1974	DQ380163
*Aedes cuminsi* mosquito	ArD38388	Burkina Faso	1983	DQ380181
*Hipposideros caffer* bat	ANK3837	Guinea	1981	DQ380165
*Micropterus pusillus* bat	ANK6087	Guinea	1984	DQ380166
Human	CARR1622	Central African Republic	1985	DQ380160
*Eretmapodites* sp. mosquito	Entebbe	Uganda	1944	DQ380156
Human	HvB375	Central African Republic	1985	DQ380161
Bovine	KenyaIB8	Kenya	1965	DQ380176
*Ae. macintoshi* mosquito	Kenya21445	Kenya	1983	DQ380171
Human	Kenya00523	Kenya	1998	DQ380169
Human	MgH824	Madagascar	1979	DQ380144
Human	OS1	Mauritania	1987	DQ380180
Human	OS3	Mauritania	1987	DQ380178
Human	OS8	Mauritania	1987	DQ380177
Human	OS9	Mauritania	1987	DQ380179
Ovine	SA51	South Africa	1951	DQ380158
Human	SA75	South Africa	1975	DQ380175
Human	Saudi10911	Saudi Arabia	2000	DQ380170
Bovine	ZC3349	Egypt	1978	DQ380152
Human	ZH501	Egypt	1977	DQ380149
Human	ZH548	Egypt	1977	DQ380151
Human	ZH1776	Egypt	1978	DQ380153
Mosquito	ZM657	Egypt	1978	DQ380146
Ovine	ZS6365	Egypt	1979	DQ380145
Human	Zinga	Central African Republic	1969	DQ380167
Bovine	0611MeruSouth	Kenya	2007	EU574078
Bovine	0094Garissa	Kenya	2007	EU574086
Buffalo	2820Garissa	Kenya	2007	EU574061
Human	2008/00099	Mayotte	2008	HE687302
Human	2008/00101	Mayotte	2008	HE687307
Bovine	An1000	Madagascar	1991	EU312108
Human	3162	Madagascar	2008	JF311386
Human	3163	Madagascar	2008	JF311387
Human	3164	Madagascar	2008	JF311388
Human	3165	Madagascar	2008	JF311389
Bovine	3168	Madagascar	2008	JF311392
Bovine	3169	Madagascar	2008	JF311393
Bovine	3170	Madagascar	2008	JF311394
Human	SPU10315	Kenya	2007	EU312147
Human	SPU384001	Kenya	1997	EU312128
Human	B1143	Kenya	1977	EU312119
Goat	1811Garissa	Kenya	2006	EU574068
Bovine	1602Mombassa	Kenya	2007	EU574071
Ovine	473Kajaido	Kenya	2007	EU574080
Ovine	3644Baringo	Kenya	2007	EU574059
Human	004/006Garissa	Kenya	2006	HM586975
Human	035/07Barissa	Kenya	2007	HM586980
*Aedes* sp. mosquito	131B04/06Garissa	Kenya	2006	HM586983
*Aedes* sp. mosquito	KLF091/07	Kenya	2007	HM586984
Human	Tanga001/007	Tanzania	2007	HM586981
Human	Dodoma002/07	Tanzania	2007	HM586982
Human	HB29 Phlebovirus	China	2010	HM745932
